# Alternative Energy: Breaking Down the Diverse Metabolic Features of Lung Cancers

**DOI:** 10.3389/fonc.2021.757323

**Published:** 2021-10-21

**Authors:** Kasey R. Cargill, William L. Hasken, Carl M. Gay, Lauren A. Byers

**Affiliations:** Department of Thoracic/Head and Neck Medical Oncology, University of Texas MD Anderson Cancer Center, Houston, TX, United States

**Keywords:** lung cancer, metabolism, metabolic inhibitors, glycolysis (Warburg effect), oxidative phosphorylation

## Abstract

Metabolic reprogramming is a hallmark of cancer initiation, progression, and relapse. From the initial observation that cancer cells preferentially ferment glucose to lactate, termed the Warburg effect, to emerging evidence indicating that metabolic heterogeneity and mitochondrial metabolism are also important for tumor growth, the complex mechanisms driving cancer metabolism remain vastly unknown. These unique shifts in metabolism must be further investigated in order to identify unique therapeutic targets for individuals afflicted by this aggressive disease. Although novel therapies have been developed to target metabolic vulnerabilities in a variety of cancer models, only limited efficacy has been achieved. In particular, lung cancer metabolism has remained relatively understudied and underutilized for the advancement of therapeutic strategies, however recent evidence suggests that lung cancers have unique metabolic preferences of their own. This review aims to provide an overview of essential metabolic mechanisms and potential therapeutic agents in order to increase evidence of targeted metabolic inhibition for the treatment of lung cancer, where novel therapeutics are desperately needed.

## Introduction

Lung cancer continues to be recognized as the leading cause of cancer-related deaths in the United States (1). Non-small cell lung cancer (NSCLC) accounts for around 85% of all lung cancers and includes adenocarcinoma (40-50%), squamous cell carcinoma (SCC; 25-30%), and large cell carcinoma (3-10%) (2, 3). Approximately 25% of these tumors are diagnosed early in disease progression when surgical resection is the primary treatment leaving them with a 60% five year survival rate (2, 4). Unfortunately the remaining diagnoses are ineligible for surgery due to advanced disease and receive frontline chemotherapy or radiation and have a five year survival rate of 23% (2). In comparison, SCLC accounts for 15% of all lung tumors, but has a substantially lower five year survival rate of only 7% (1). SCLC is not routinely resected due to frequently advanced staging at the time of diagnosis, therefore despite recent advances in chemo- and immunotherapies, prognosis remains poor. The dismal survival rates and rapid relapse among all types of lung cancer, highlights the importance of research into personalized therapeutic strategies.

Many cancer investigations have underscored the significance of altered metabolic phenotypes in both the tumor and tumor microenvironment (TME), however few studies in lung cancer (both NSCLC and SCLC) have been aimed at understanding the contribution of metabolic dysregulation to disease progression and therapy response. This review aims to provide an overview of essential metabolic mechanisms and potential therapeutic agents in order to increase evidence of targeted metabolic inhibition for the treatment of lung cancer.

## Lung Cancer as a Metabolic Disease

**Figure 1:**
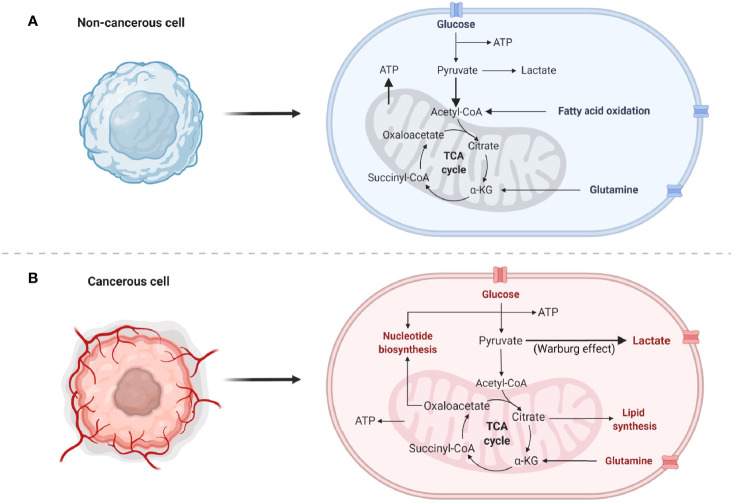
Normal and cancer cell metabolisms. **(A)** Common normal, non-cancerous metabolic pathways predominantly utilized by the cells. Bold arrows indicate increased flux of pyruvate into the mitochondria for the generation of ATP. **(B)** Highly proliferative cancer cell metabolism utilizes numerous pathways to generate energy, nucleotides, lipids, and amino acids. Bold arrow indicates preferential conversion of pyruvate to lactate, known as the Warburg effect.

Cancer metabolism has been a prominent avenue of investigation since the 1920s when Dr. Otto Warburg classified what is now known as the Warburg effect (5, 6). This observation that cancer cells exhibit enhanced glucose metabolism over the more efficient oxidative metabolism became a hallmark of the disease and is still a widely accepted and investigated phenomenon. The Warburg effect is comprised of three main aspects: 1) enhanced glucose uptake 2) increased lactate secretion and 3) decreased oxidative metabolism (**Figure 1**) (7–9). Dr. Warburg originally attributed the decrease in oxidative metabolism to mitochondrial dysfunction; however this hypothesis has since been disputed. While some tumors do exhibit loss of mitochondrial density or altered dynamics rendering the organelle non-functional, other types retain their oxidative metabolic capacity entirely and may even up-regulate oxidative mechanisms of nutrient production, particularly in chemoresistant tumors (8, 10). This suggests that cancer cells are adaptive in terms of the metabolic pathways needed for tumorigenesis and cancer persistence. Therefore, today it is realized that each cancer needs to be independently evaluated for metabolic pathway utilization. These crucial differences in the metabolic preference of cancer are at the forefront of investigation and may hold the key to identifying novel molecules for therapeutic targeting with broad application to the personalization of cancer medicine.

### Metabolic Pathways Contributing to Cancer

Increased aerobic glycolysis characterized by uptake of glucose and lactate secretion is the most notable effect described by Warburg (7, 8, 11). This phenomenon is observed in many cancers, however the mechanisms driving this phenotype are significantly more complex. For example, several oncogenic pathways have been implicated in the up-regulation of glycolysis, including MYC, PI3K-Akt-mTOR, and stabilized HIF-1/2α to name a few (**Table 1**) (8, 29–31). Collectively, these pathways are involved in increased expression of almost every enzyme in the glycolysis pathway. More recently, mechanisms such as HIF stabilization have been shown to concurrently down regulate mitochondrial pyruvate oxidation (32, 33). Other such mechanisms also exist including regulation through reactive oxygen species (ROS) (34). Apart from suppression of mitochondrial respiration, several reports have revealed that mitochondria may also be crucial for energy and biosynthetic precursor generation as well (35–37), but this has been less readily interrogated. Because of the complexity and differences of metabolic regulation in cancer cells, an emerging hypothesis is that the metabolic profiles of individual cancer cells may be as heterogeneous as the tumor itself.

**Table 1:** The effect of genetic mutation on metabolism.

Gene Mutation	Expression Change	Altered Pathway	References
EGFR	↑	Glycolysis Nucleotide metabolism	Jin et al. (12); Bethune et al. (13)
KEAP1	↓	Glutaminolysis	Romero et al. (14)
KRAS	↑	Fatty acid metabolism GlycolysisPPP	Jin et al. (12); Pupo et al. (15); Jančík et al. (16); Padanad, et al. (17)
LKB1	↓	Glutaminolysis	Galan-Cobo et al. (18)
MYC	↑	Fatty acid metabolism Glutaminolysis Glycolysis	Chalishazar et al. (19); Rapp et al. (20); Marengo et al. (21)
NOTCH1	↑	Glutaminolysis GlycolysisOxidative phosphorylation	Sellers et al. (22); Zou et al. (23)
NTRK1	↑	Glutaminolysis GlycolysisOxidative phosphorylation	Vaishnavi et al. (24); Yang et al. (25)
P53	↓	Glycolysis	Jin et al. (12)
PTEN	↓	Glycolysis	Jin et al. (12); Georgescu (26)
RB1	↓	Amino acid metabolism GlycolysisNucleotide metabolism	Bhateja et al. (27); Mandigo et al. (28)

Lung cancers typically acquire specific genetic mutations leading to tumor formation and progression. Several commonly mutated genes lead to metabolic changes that result in therapy resistance. ↑ indicates increased expression; ↓ indicates decreased expression.

Metabolic reprogramming is just one hallmark of cancer that serves to facilitate rapid cellular proliferation, avoidance of cell death, and mitigation of stress responses. Although non-cancerous, terminally differentiated cells rely on oxidative phosphorylation (OXPHOS) to meet energy demands, cancer cells require nucleotides [generated by the pentose phosphate pathway (PPP)], reducing equivalents [generated by glycolysis, the PPP, and the tricarboxylic acid cycle (TCA)], and amino acids (taken in from the TME or generated predominantly from the PPP or TCA) in addition to energy produced from glycolysis and OXPHOS to adapt to constant changes in their environment (**Figure 1**). See the following review for an in-depth overview of the aforementioned metabolic pathways in lung cancer (38). In addition to these biomolecules supporting tumor growth, many metabolites also play a role in anti-apoptotic signaling and interaction with the TME (39, 40). The current literature depicting the metabolic processes provides insight into why lung cancers exhibit aggressive tumor growth, making it the number one cause of cancer-related deaths (1, 41).

### Non-Small Cell Lung Cancer

Although the morphological and genetic components leading to NSCLC are largely known, long-term survival of disease remains inadequate despite recent advances in personalized treatment and immunotherapies (42, 43). Thus, recent studies have been aimed at elucidating the metabolic properties and vulnerabilities driving NSCLC (44). Unlike some cancers that exhibit clear-cut dependence on a particular metabolic pathway, NSCLC utilizes multiple pathways to drive proliferation (44)—however it is unclear whether these pathways operate simultaneously or arise due to the heterogeneous cell population found in the diverse tumor environment. Studies investigating the mechanisms that dictate tumor growth have shed light on the importance of cellular metabolism in driving disease and have become the focus of several therapeutic opportunities (17, 18). While these reports show metabolic reprogramming is a contributor to cancer, few treatment options have progressed through early stage clinical trials despite promising pre-clinical results. In NSCLC specifically, recurrently mutated oncogenes and tumor suppressors (*TP53*, *EGFR*, *KEAP1*, and others) have been implicated as regulators of metabolism and major drivers of metabolic reprogramming (**Table 1**) (44).

To determine whether metabolic heterogeneity is related to increases in both glycolysis and TCA cycle intermediates, one study profiling 80 NSCLC human cell lines found that the ratio of glucose utilization and lactate secretion varied greatly between samples indicating that the Warburg effect is not a universal characteristic of NSCLC (44). In fact, NSCLC can be divided into at least glycolysis-dependent and OXPHOS-dependent subtypes (45). NSCLC cell lines subjected to Seahorse extracellular flux analysis treated with either metformin (OXPHOS inhibitor) or a MCT4 (lactate) inhibitor found that OXPHOS-dependent cells were sensitive to metformin, whereas cellular proliferation was attenuated by MCT4 inhibition specifically in the glycolysis-dependent cells (45). Other investigations have shown that NSCLC cells also take in lactate through MCT1 lactate transporters to utilize as a carbon source in the TCA cycle and lipid biosynthesis (36, 38, 46). This suggests that an increased flux through glycolysis may directly supply lactate for paracrine reuptake to meet both aerobic and anaerobic cellular demands. Although cell lines are a valuable tool for investigating the complexities of metabolism, the differences between immortalized cell lines and primary resected tumors adds difficulty to teasing apart metabolic discrepancies between studies.

Several studies have interrogated the cellular and genetic discrepancies among the most common subsets of NSCLC—adenocarcinoma and squamous cell carcinoma (SCC) (2), therefore is likely that there are metabolic differences as well. Resected human adenocarcinoma and SCC tumors subjected to stable isotope tracing indicated that squamous cell carcinoma relies on NOTCH1-driven glucose and glutamine catabolism to a greater extent than adenocarcinoma, suggesting enhanced glycolysis is a crucial driver for the quick progression of SCC (22, 47). A 24-gene signature comprised of glycolysis (ALDOC, GAPDH, PGAM, and TPI), PPP (G6PDH and TALDO1), nucleotide synthesis (CTPS1, GMPS, PAICS, and UMPS), amino acid biosynthesis (AHCY, ASNS, BDH1, CKMT1, GCLM, GGH, GSS, MTHFD2, PSAT1, and SHMT2), and TCA cycle (GOT2, IDH2, MDH2, and ME1) genes was elucidated between SCC and adenocarcinoma and conferred a worse outcome in SCC patients (22). In addition to gene expression, enzymatic activity was enhanced across 10 glycolytic enzymes in SCC compared to adenocarcinoma, which correlated to the NOTCH pathway (including MYC expression) (22). Interestingly, in addition to glycolysis, TCA cycle intermediates, fatty acid synthesis biomolecules, and reducing equivalents were all increased in SCC, however it is hypothesized that this is to regenerate NAD+ for glycolysis (22). Another explanation is that NSCLC has uniformly enhanced bioenergetics or more likely, it is comprised of glycolytic and oxidative regions that are challenging to delineate and will require more sophisticated single cell analysis.

Other recent clinical work performed ^13^C-glucose diffusion in nine NSCLC patients and found an increase in glucose and TCA-derived metabolites (i.e. lactate, citrate, glutamate, and malate) (48). Of these patients, four had EGFR mutations, two harbored KRAS mutations, and the remaining three did not have either mutation (48). The group further showed that neither mutation status conferred unique metabolic alternations (48). Although mutational status was not predictive of the exact metabolic changes that would be induced in a patient, which may in part be due to the small sample size, these mutations are quite common among NSCLC. In fact, lung adenocarcinoma can be classified by genetic mutations in *TP53* (46%), *KRAS* (32%), *EGFR* (27%), and *KEAP1* (23%), among others (14, 49, 50) and SCC may have mutations in *TP53* (90%), *KEAP1* (31%), and *PTEN* (15%) and others (**Table 1**) (50–52). Further, these mutations may provide insight into the metabolic state of each cancer type.

*TP53* mutations, implicated in both adenocarcinoma and SCC, have profound significance in altering metabolism. Wild type p53 plays a role in maintaining OXPHOS by assembling complexes of the electron transport chain while simultaneously inhibiting glycolytic enzyme transcription and the oxidative branch of the PPP (38). In line with these observations, p53 expression has been identified as a biomarker of resistance to the glycolysis inhibitor 2-deoxy-D-glucose (2DG) such that p53-deficient NSCLC cells (H358) exhibit significantly reduced ATP levels accompanied by profound oxidative stress when treated with 2DG (53) suggesting that glycolysis inhibition would be preferentially beneficial in tumors lacking p53.

*EGFR* mutations occur most often in lung adenocarcinomas and play an important part in mediating global metabolic reprogramming. Alterations in *EGFR* commonly result in the Warburg effect through stabilization of glucose transporters. Further, signaling through the PI3K/AKT/mTOR pathway promotes glycolysis by regulating the localization of glucose transporter GLUT1 to the plasma membrane in *EGFR*-mutated NSCLC (54). Moreover, glutaminolysis is increased and inhibition with erlotinib in combination with CB-839 (glutaminase inhibitor) in *EGFR*-mutated tumors resulted in tumor regression (55). This sets precedence for combinatorial approaches targeted at altered metabolism and genetic mutations in lung cancer.

*KRAS* activating mutations are common and mutually exclusive to *EGFR* mutations. *In vivo* lung tumors with depleted KRAS exhibit reduced glycolysis and lipid gene expression leading to reduced uptake of these associated metabolites, consistent with reports that show KRAS overexpression up-regulates these pathways (17, 56). Further, inhibition of the glycolysis pathway with 2DG in KRAS mutant NSCLC models significantly attenuated cell line and tumor growth (57). Because the mutant form of KRAS has thus far been untargetable by conventional chemotherapeutic agents, it is advantageous to identify targets enhanced by this *KRAS* mutation (51). To this affect, studies have been aimed at investigating targetable mechanisms downstream of the mutation, including the consequential metabolic reprogramming that occurs. This serves as yet another example of how targeting major metabolic pathways may lead to treatment options capable of reducing tumor growth regardless of mutation status.

*KEAP1* mutations often occur concurrently with *KRAS* mutations in adenocarcinomas, however can occur independent of *KRAS* particularly in SCC (14, 58). Although *KRAS* mutant tumors are largely characterized by glucose and lipid metabolizing pathways, *KEAP1* mutations are also highly dependent on glutamine (14). This glutamine dependence has been therapeutically targeted with CB-839 in lung adenocarcinoma xenografts which revealed decreased tumor growth rates (14). Interestingly, *KEAP1* loss also decreases the production of ROS and enhances resistant to oxidative stress (58). This is through the regulation of NRF2 protein stability, a mediator of pathways including cellular stress, autophagy, proliferation, and metabolism. Together, KEAP1/NRF2 coordinate to reprogram cancer cells towards pathways that support glycolysis, mitochondrial respiration, and amino acid biosynthesis (14, 59).

Lastly, *LKB1* inactivation or mutation occurs in nearly 20% of NSCLC cases, and, similarly to KEAP1 mutations, occur concurrently with KRAS mutations in 7-10% of NSCLCs (60). LKB1 canonically phosphorylates the family of AMP-related kinases, which are major sensors of cellular energy that target mitochondria and fatty acid metabolism pathways. Due to this, LKB1-deficient lung cancer cells were preferentially susceptible to the mitochondrial electron transport chain complex I inhibitor phenformin (60). This effect was not seen with the similar agent metformin nor the glycolysis inhibitor 2DG, due to an induction of ROS leading to increased mitophagy (60). In addition to LKB1 and KRAS concurrent mutations, KEAP1 inactivating mutations are also often enriched for simultaneous KEAP1 mutations. Collectively, these three mutations cooperatively drive dependence on glutamine and thus, are sensitive to CB-839 *in vitro* and *in vivo* (18). These data indicate that LKB1 mutations do not reprogram towards glycolysis and instead are reliant on OXPHOS to drive tumor progression.

Studies such as these, that elucidate the contribution of mutational status to metabolic rewiring, lay the foundation for use of metabolic modulators in NSCLC. Although, it is clear that additional phenotyping across cell lines and primary tumors is required to identify biomarker predictors of metabolic pathway utilization. In addition to this, it is likely that metabolic signatures of tumors may be regulated by addition mechanisms including DNA methylation (44). It is evident that there are numerous contributors to cellular metabolism. Much like the genetic heterogeneity seen in solid tumors, there is growing evidence that NSCLCs exhibit localized regions in the tumors that may have different nutrient requirements, which may be dependent on various factors including nutrient availability, oxygenation, and immune infiltration (48).

### Small Cell Lung Cancer

Unlike NSCLC, SCLC is characterized by universal loss of *RB1* and *TP53* and traditionally diagnosed, classified, and treated as a single disease (**Table 1**) (51, 61–64). The evolution of SCLC subtyping has occurred over the past 30 years starting with the observation that SCLC cell lines had two prominent biochemical signatures, which resulted in classification of *classical* and *variant* subtypes (65). Moreover, the variant subtype was further divided into categories dependent on unique biochemical, morphological, and growth properties (66). Once this initial characterization was established, several studies began looking at the unique molecular signatures (67, 68), which included the identification of the neuroendocrine transcription factor subtypes ASCL1 (67, 69, 70) and NEUROD1 (67, 79), the non-neuroendocrine, tuft-cell variant classified by POU2F3 (67, 71), *MYC*-driven populations (19, 68), and *YAP/TAZ* variant phenotype (67, 72). Although much effort has been directed towards finding an appropriate characterization system, less is known about the metabolic preferences and pathway utilization, which may further delineate SCLC.

SCLC is most notably characterized by loss of *RB1* and *P53*, both of which regulate various metabolic pathways (**Table 1**) (28, 73–75), therefore the observation of metabolic differences based on these alone would not provide unique and targetable pathways. Metabolically, the most well studied subcategories of SCLC are driven by ASCL1 and MYC expression. ASCL1 is a transcription factor dictating neuroendocrine lineage that can be stratified into ASCL1^high^ and ASCL1^low^ populations (76). Interestingly, ASCL1^low^ cell lines and tumors often highly express the transcription factor MYC, which is implicated in approximately 20% of SCLC (68, 73, 76). The ASCL1^Low^/MYC^High^ phenotype also typically has high NEUROD1 [in cell lines and genetically engineered mouse models (GEMMs)] or POU2F3 (in patient tumors) expression, however this discrepancy between cell lines, mouse models, and patient tumors is not well understood (73, 74, 77, 78).

Combined metabolic and transcriptional profiling of a panel of 29 SCLC cell lines and 47 primary SCLC tumors revealed that *ASCL1* was the top differential gene delineating two major metabolomics profiles (76, 79). The identified metabolites were linked to nucleotide biosynthesis, amino acid metabolism, and the TCA (76). Interestingly, several purine, but not pyrimidine, nucleotides were significantly elevated only in the ASCL1^Low^ cell lines (76). Similarly, transcriptional data from 81 patient tumors (74) revealed that genes linked to purine synthesis (*IMPDH1* and *IMPDH2*) were also enriched in approximately 20% of the tumors that also had low *ASCL1* expression (76). Moreover, *MYC* expression strongly correlated with *IMPDH1* and *IMPDH2* and ChIP-seq experiments confirmed direct MYC binding to the promoter region of these genes (76). This led to a hypothesis that IMPDH may be a targetable biomolecule and CRISPR/Cas9 *IMPDH1* knockdown and treatment with the IMPDH inhibitor mycophenolic acid (MPA) both lead to significant decreases in cellular viability in treatment-naïve and chemoresistant SCLC (76, 79). Clinically, this provides a basis for investigation into the use of IMPDH inhibitors such as MPA and mizoribine, but also may in part explain why anti-folates and nucleoside analogues are moderately successful in NEUROD1 and POU2F3-expressing SCLC, which commonly exhibit MYC overexpression (78, 80).

In addition to nucleotide synthesis, the ASCL1^Low^MYC^High^ phenotype has also been implicated in alterations in amino acid and polyamine synthesis in SCLC (19, 76). Tumors from ASCL1-driven Rb1^fl/fl^;p53^fl/fl^;Pten^fl/fl^ (RPP) mice and MYC-driven (NEUROD1 phenotype) Rb1^fl/fl^;p53^fl/fl^;MycT58A^LSL/LSL^ mice exhibit metabolically distinct patterns with particular enrichment in the arginine and proline biosynthesis pathways (19). In line with this, inhibition of polyamine biosynthesis with NOS, ODC1, or mTOR inhibitors and siRNAs against *ODC1* reduced cellular proliferation and viability in MYC-driven SCLC cell lines (19). Moreover, metabolic distinctions between treatment-naïve and chemo-resistance revealed that chemo-resistant cell lines exhibited a dependence on arginine and polyamine biosynthetic pathways as well as the mTOR pathway that was directly modulated by MYC expression (19). Not only does MYC play a key role in the metabolic phenotype of SCLC, but also in the evolution of the molecular subtype profile (19). MYC has been shown to regulate the dedifferentiation of ASCL1+ neuroendocrine cells through promotion of Notch signaling to support the evolution of NEUROD1+ and YAP1+ cells (19). While MYC has not been directly implicated in the emergence of chemo-resistance, MYC-driven fluctuations in Notch signaling activation and metabolic alterations may contribute to the plasticity of SCLC subtypes and appearance of subtype evolution or tumor heterogeneity (19, 78, 81, 82)

Nucleotides and amino acids are essential for the rapid proliferation that characterizes cancer; however, the specific pathways that generate these biomolecules are relatively understudied in SCLC. With the recent introduction of molecular subtyping and the initiative to discover subtype-specific therapies, metabolic profiling may offer valuable insight into new therapeutic targets. Although, current pathway analysis is limited, Morita et al. performed an investigation into the role of the glycolysis enzymes PKM1 and PKM2 in neuroendocrine SCLC (80). PKM1 is often expressed in terminally differentiated cells, while PKM2 is more commonly expressed by proliferating cells and cancer regulated by MYC (83). PKM2 is therefore likely favored by cells exhibiting the Warburg effect, whereas PKM1 is preferred by more oxidative tumors in most cases (84, 85). In a pan-cancer analysis, the PKM1/PKM2 ratio was higher in SCLC compared to several other types of cancer, however it is important to note that PKM1 was still not the major PK isoform expressed (only 16-38%) (80). PKM1 was also found to be required for PKM2 activation leading to cellular proliferation and exclusive expression of PKM1 facilitated active flux of glucose-derived carbons into the TCA with reduced lactate production (80). With the regulation of both glucose catabolism and OXPHOS by PK isozymes, inhibitors of these crucial pathways may prove effective. Unfortunately, there are no current investigations into the use of glycolysis inhibitors, however a Phase II clinical trial with CP-613 has been conducted in a small cohort of 12 patients with relapsed SCLC (86). CP-613 is a lipoate analogue that targets pyruvate dehydrogenase (PDH) and alpha-ketoglutarate dehydrogenase (KGDH), two key mitochondrial enzymes. Although efficacy was poor with no partial or complete responses, all 3 patients who subsequently were treated with topotecan exhibited robust response (86). Moreover, *in vitro* combination of CP-613 with topotecan was synergistic and offers evidence for a combinatorial approach of metabolic inhibitors and chemotherapy in future investigations (86).

Large cell neuroendocrine carcinoma (LCNEC) is a rare form of lung cancer (approximately 3%) associated with *TP53* (86%) and/or *RB1* (36%) gene alterations (**Table 1**) (3, 87). While LCNEC is classified as a variant of NSCLC, the transcriptional properties and clinical treatment regimen is quite similar to SCLC (3, 88). An integrative genomic and transcriptomic profiling of LCNEC revealed two subclasses: Type I (ASCL1^High^/DLL3^High^/Notch^Low^) and Type II (ASCL1^Low^/DLL3^Low^/Notch^High^). Type I LCNEC shared closest similarities with classic ASCL1-driven SCLC and exhibited increased expression of genes involved in energy generation, OXPHOS, ETC/ATP synthase pathways (88). This suggests that ASCL1-driven SCLC and Type I LCNEC are more reliant on mitochondrial respiration rather than the Warburg effect (88). While we can extrapolate that Type II LCNEC is more similar to variant NEUROD1- or MYC-driven SCLC, further metabolomic profiling is required.

## Nutrient Competition and the Tumor Microenvironment

**Figure 2:**
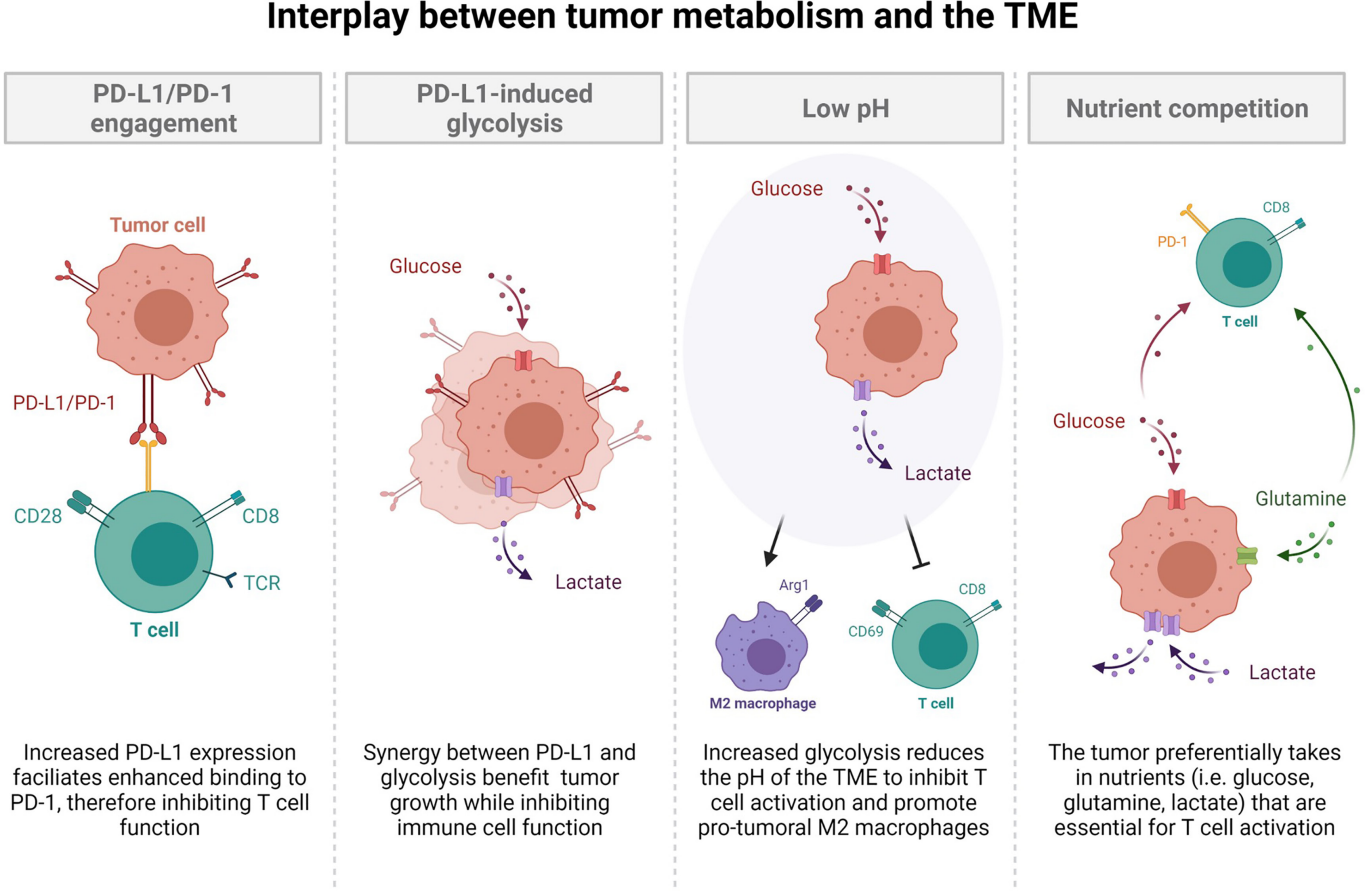
The interplay between tumor metabolism and the tumor microenvironment. Increased PD-L1 expression facilitates enhanced binding to PD-1, thereby inhibiting T cell function. Synergy between PD-L1 and glycolysis benefit tumor growth while inhibiting immune cell function. Increased glycolysis reduces the pH of the tumor microenvironment to inhibit T cell activation and promotion of pro-tumoral M2 macrophages. The tumor preferentially takes in nutrients such as glucose, glutamine, and lactate, which are all essential for T cell activation. This nutrient competition leaves resources scarce for optimal T cell activation.

With the recent emergence of immunotherapies (specifically, immune checkpoint blockade; ICB) and their usage in both NSCLC and SCLC, it is crucial to understand the role of metabolism in the regulation of an immune response in cancer. As previously demonstrated in this review, therapeutics for cancer have the ability to alter cellular metabolic programs used by cancer cells. Understanding the metabolic changes that occur as a result of therapy may shed light on new opportunities for combinatorial treatments that are more beneficial than front line therapies. Importantly, immune cell activation, expansion, and function require the same nutrients and metabolic pathways as cancer cells, with a specific dependence on glycolysis (40). Since tumor cells are often highly glycolytic, they outcompete immune cells for glucose, amino acids, and fatty acids leading to immune dysfunction and an inability to clear tumor antigens (**Figure 2**). This nutrient competition has also been implicated in driving tumor progression (40, 89). In addition to hoarding glucose, cancer cells have the unique ability to evade the immune system *via* metabolite secretion (lactate) and expression of immune checkpoint molecules (PD-L1), both of which decrease immune cell cytokine production (IFN-γ), glycolysis, and immune cell expansion (**Figure 2**) (90). This environment favors cancer persistence and leads to decreased immune cell function while promoting an anti-inflammatory environment that confers tolerance to the growing tumor.

The major product of the Warburg effect, lactate, is secreted into the tumor microenvironment (TME) by rapidly proliferating tumors (3, 36, 46, 91). This serves to acidify the TME region, which 1) fuels mitochondria 2) suppresses the immune system and 3) promotes metastasis through therapy resistance (**Figure 2**) (91). The metabolic heterogeneity of tumors allows for glycolytic and oxidative cells to work symbiotically through a bidirectional pyruvate to lactate conversion (91). As previously discussed in NSCLC, glycolytic cells secrete lactate through MCT4 while oxidative cells uptake lactate through MCT1 (46, 91), which maintains an acidic TME while providing fuel for *de novo* amino acid, nucleotide, and fatty acid synthesis. Moreover, a result of oxidative metabolism is ROS, which act as signaling molecules to suppress immune function (92, 93). More directly however, a decreased pH, due to lactate secretion, augments signaling pathways of immune cells, rendering them incapable of efficient activation through the down regulation of glycolysis-promoting mechanisms, leading to T cell exhaustion, apoptosis, and a pro-tumoral M2 macrophage phenotype (**Figure 2**) (91).

Current investigations have examined the efficacy of ICB as a single agent and in combination with chemotherapy and glycolysis inhibitors and found that glycolysis inhibition does not negatively affect immune function, since these drugs are taken in most rapidly by glucose-addicted cancer cells (94). Together, metabolism and nutrient availability are important factors that dictate the microenvironment’s ability to promote immune evasion and tumor progression. In addition to altering the metabolic reprogramming required for proper immune cell activation, many tumors, including NSCLC and SCLC, express immune checkpoint molecules, such as PD-L1 (89). PD-L1-expressing tumor cells engage with PD-1 on lymphocytes to actively suppress immune cell expansion and effector function (**Figure 2**) (89). Apart from this, PD-L1 and glycolysis have been shown to be positively correlated, although it is currently unclear whether PD-L1 expression enhances glycolysis or vice versa. One study has shown that glucose deprivation lead to an up-regulation of PD-L1, while siRNA knockdown of PD-L1 likewise decreased expression of glycolysis enzymes (specifically PFKFB3) in NSCLC cell lines (95). Further, another investigation found that PD-L1 increased expression of the glycolysis enzyme HK2 in SCC NSCLC (96). These studies suggest that PD-L1 may be directly involved in the up-regulation of glycolysis and elude to potential signaling mechanisms such as PI1K/AKT/mTOR, EGFR, and HIF-1α (95–98). Interestingly, other investigations have concluded that the metabolic switch towards glycolysis is essential for PD-L1 overexpression (99, 100). Regardless of the mechanisms leading to synergy between glycolysis and PD-L1, it is clear that their up-regulation facilitates immune dysfunction and is associated with poorer survival (99, 101). Due to this, it would be interesting to investigate the ability of PD-L1 to be a potential biomarker of highly glycolytic tumors, which would allow for metabolic intervention with inhibitors of the glycolysis pathway. While use of ICBs have variable success in the clinic, combinatorial therapies utilizing frontline chemotherapy and ICB plus glycolysis inhibitors may be more effective to restore the nutrient balance in the TME and promote reinvigoration of the immune system to promote tumor clearance.

## Metabolic Implications of Therapy in Lung Cancer

### Fueling Resistance: Metabolic Alterations and Standard of Care

Current frontline efforts aimed at mitigating lung cancer is highly dependent on the subtype of disease and stage of progression at the time of diagnosis. Regardless of the treatment regimen, the baseline metabolic profile of the tumor plays a role in therapy sensitivity and rate of relapse. Cisplatin (a common platinum-based chemotherapy) resistance in particular is thought to be a result of lung cancer with a more oxidative phenotype, characterized by increased mitochondrial density, ROS, and dependence on glutamine and fatty acid oxidation mechanisms (102–105). Carboplatin (another platinum-based chemotherapy) resistance, however is associated with a greater dependence on glycolysis, possibly mediated by MYC expression (102, 106, 107). It is unclear whether these profound differences in resistance mechanisms are due to metabolic reprogramming events triggered by the treatment or whether the treatment selectively targets cells utilizing specific pathways from a metabolically heterogeneous population.

The recent approval of immunotherapies to be used as a standard of care has offered benefit to only subsets of patients (108). Understanding the role of immunotherapy in altering both tumor and immune metabolisms could provide key insights into why the rates of relapse for NSCLC and SCLC have not dramatically changed since this advancement. As previously discussed, the tumor and the immune system are in constant competition for access to the essential nutrients required for expansion of both cell populations. Therefore, optimal inhibition would block nutrient flux into the tumor leaving the essential molecules in the TME for immune activation. The addition of immunotherapy enhances mitochondrial activity and ROS production in tumor cells, which serves to divert glucose to the immune cells, and thus promoting activation unless terminal exhaustion has been attained (40, 109). ROS, however, can act as a double- edged sword for the immune system. While the canonical role is often associated with cytotoxic capabilities and promotion of DNA damage, another emerging role for ROS is as critical secondary messengers important for T cell differentiation and function (110). Metabolically, low to moderate levels of ROS are required for T cell metabolic reprogramming towards aerobic glycolysis upon T cell activation, and use of a manganese metalloporphyrin (ROS scavenger) significantly reduced function and engagement in the glycolysis pathway (111). In the TME, similar studies suggest that ROS levels exceed an advantageous amount, therefore a strict balance is required for inducing T cell activation without causing functional inhibition (110). Although ICB attempts to facilitate immune activation, infiltration into large tumor masses often remains futile due to high ROS levels, lack of proper nutrients, and an acidic environment. This necessitates additional management of tumor growth and metabolic inhibitors would be a prime course of action. In fact, ongoing studies have seen improvement in ICB intervention with the addition of glycolysis, metabolite, and OXPHOS inhibitors in pre-clinical investigations (109, 112, 113).

### Antimetabolites as Anticancer Drugs

While a portion of lung cancers have been meticulously characterized by alterations in gene expression and oncogene/tumor suppressor mutations, there has been little progress in developing therapies that target these mutations and effectively achieve adequate therapeutic outcomes in all patients. Because of this, it may be beneficial to explore treatment options that target the accelerated DNA replication that occurs in lung cancer cells. First employed clinically in the 1940’s by Dr. Sidney Farber, antimetabolites work by mimicking substrates to irreversibly inhibit enzymes needed for DNA replication (114, 115). The effects of antimetabolites are generally cytotoxic, conferring the most pronounced effects on cells that are most metabolically active (116). While this class of drugs was originally used to treat lymphoblastic leukemia in children nearly a century ago, the use of antimetabolites as broad anti-cancer drugs did not achieve substantial popularity until much more recently (114, 115). What started as a single class of synthetic folate analogues has since expanded to a much broader collection of drugs targeting a larger array of enzymes essential to cellular metabolism. Two agents – gemcitabine and pemetrexed – are examples of antimetabolites used clinically today that may give way to new, more efficacious therapies in lung cancer. These antimetabolites, if any, may bear more exploration.

The nucleoside analogue gemcitabine is a potent pyrimidine antimetabolite that has historically been used as a first-line therapy for pancreatic adenocarcinoma, but has also been used to treat solid tumors in patients with breast, ovarian, and lung cancers (117). In its active form, gemcitabine interferes with cellular metabolism by acting as a nucleoside analogue to inhibit DNA synthesis (117). Gemcitabine has been particularly useful as an anti-cancer therapy because of additional effects that preferentially stimulate apoptotic signaling pathways in malignant cells through caspase activation. While this treatment offers a seemingly reliable way to target distinctly metabolically active cancer cells through restriction in DNA synthesis, literature shows chemoresistance develops quickly in a large subset of patients (117, 118). Although resistance often occurs within just weeks of initial treatment response, the mechanisms contributing to resistance are multifactorial stemming from genetic expression of the tumor and the immune cell profile. Interestingly however, a study evaluating chemoresistant SCLC patient’s response to gemcitabine exhibited an overall response rate of 13% (119). Furthermore, clinical trials in NSCLC comparing gemcitabine alone and in combination with other classic therapies have shown little difference in treatment groups (120). Together, these studies suggest that metabolic intervention to delay nucleoside biosynthesis may be most effective as a late-stage treatment for patients that have acquired resistance to front-line therapies.

Another antimetabolite that has been in use clinically over the past two decades is pemetrexed. A synthetic folate analogue akin to the drugs Farber originally employed to treat lymphoblastic leukemia, pemetrexed acts in at least three mechanisms to disrupt production of both purines and pyrimidines, thus reducing cellular proliferation. Specifically, inhibiting thymidylate synthase, dihydrofolate reductase, and GAR formyl-transferase broadly depletes folate conferring anti-tumor effects against an assortment of cancers (121). Several clinical trials have sought to discern if pemetrexed is suitable for use as a single agent or combinatorial therapy for those with NSCLC. In clinical trial, pemetrexed exhibited a significantly increased progression free survival rate compared to placebo and was relatively well tolerated by patients (122). Similarly, Karayama et al. treated chemo-naïve non-squamous NSCLC patients with either pemetrexed or docetaxel and found a significantly increased period of toxicity free survival in pemetrexed-treated patients (123). Other studies have evaluated pemetrexed in combination with platinum-based chemotherapy as front-line treatment, with no discernable added efficacy to traditional chemotherapy (124). Although pemetrexed is a common front-line therapy for lung adenocarcinoma NSCLC, resistance is common (125).

Gemcitabine and pemetrexed are just two examples of the many chemotherapeutic agents under the broad category of antimetabolites. As single agents, antimetabolites have not proven incredibly successful for the treatment of lung cancer (126), however in combination with other chemotherapy agents there is least modest improvement of efficacy *in vitro* and *in vivo* (118, 126–128). Antimetabolites that interfere with cellular metabolism by inhibiting the synthesis of the building blocks of nucleotides appears as an ideal method of slowing tumor growth. In clinical practice, however, antimetabolites require high therapeutic dosages leading to toxic side effects in some NSCLC and SCLC patients (126, 127, 129), although toxicity has been partially mitigated through the addition of chemotherapy protective drugs (129). The progress seen in clinical trials, as well as experiments with adjuvant agents that increase efficacy, offer promise for the use of antimetabolites, however further research into patient stratification and biomarkers of efficacy should be considered.

### Are Metabolic Inhibitors Effective in Lung Cancer Treatment?

**Figure 3:**
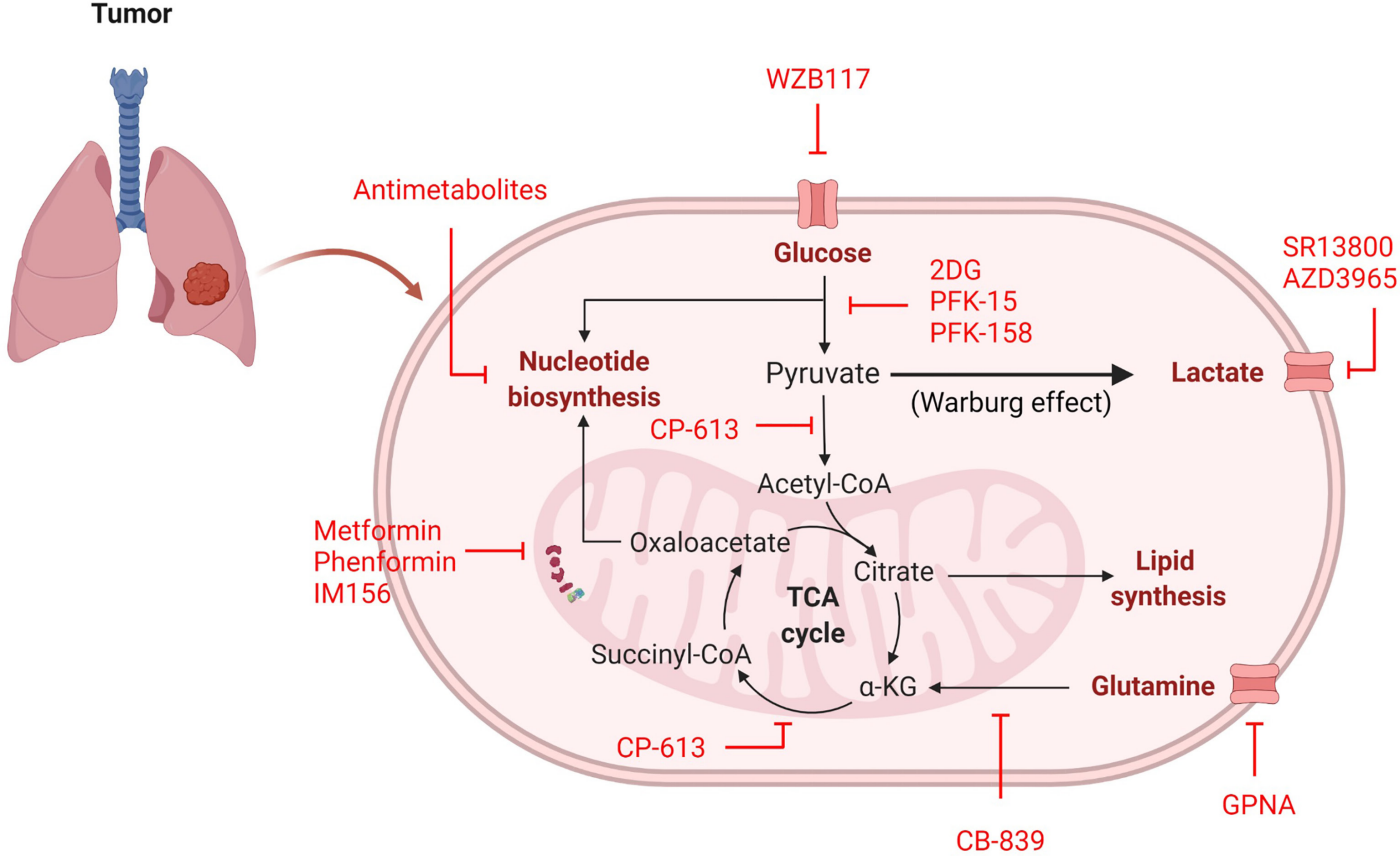
Inhibitors of cancer cell metabolism. Inhibitors (red) can target many metabolic pathways in an attempt to stop or delay energy and nutrient generation necessary for cellular proliferation.

Cellular metabolism consists of intricate pathways with the regulating molecules often rendered dysfunctional in tumors. Although signaling cascade pathways are potential therapeutic targets, toxicity in non-cancerous cells is often detrimental. To overcome this, directly modulating the metabolic pathways may prove advantageous, as the most metabolically active cells tend to be targeted by their increased uptake of nutrients—known as cellular selectivity based on demand (130). This provides several avenues for intervention by 1) stopping glucose/glutamine/lactate transport into the cell or 2) inhibiting enzymatic conversions in glycolysis and OXPHOS pathways (**Figure 3**).

Several studies have been aimed at blocking the major energy-producing carbon sources (glucose, glutamine, and lactate) from initial transport into a cancer cell (**Table 2**). Based on the predominant physiological need for glucose to support cancer cell proliferation, glucose transport inhibitors may be useful in limiting the amount of glucose taken in by the tumor. In human-derived NSCLC A549 cells, siRNA against GLUT1 inhibited colony formation, reduced proliferation, and increased apoptosis (131). When compared to non-tumorigenic lung (NL20) cells, A549 cells treated with the GLUT1 inhibitor WZB117 exhibited far less proliferation, indicating that A549 cells are inherently more glucose-dependent (**Table 2** and **Figure 3**) (132). Moreover, xenografts with A549-derived tumors that were treated with WZB117 had a 70% reduction in tumor growth compared to vehicle controls (132).

**Table 2:** Inhibitors of cancer cell metabolism.

Name of Drug	Target Pathway	Lung Cancer Clinical Trail (Clinicaltrials.gov)
AZD3965	MCT1 (Lactate transport)	NCT01791595
CB-839 (Telaglenastat)	GLS (Glutaminolysis)	NCT02771626
CD-613 (Devimistat)	Mitochondrial PDH/KGDH	N/A
IM156	Mitochondrial ETC Complex 1	NCT03272256
L-γ-Glutamyl-p-nitroanilide (GPNA)	Glutamine transport	N/A
Metformin	Mitochondrial ETC Complex 1	NCT02285855 NCT01997775
PFK-15	PFKFB3 (Glycolysis)	N/A
PFK-158	PFKFB3 (Glycolysis)	NCT02044861
Phenformin	Mitochondrial ETC Complex 1	NCT03026517
SR13800	MCT1 (Lactate transport)	N/A
WZB 117	GLUT1 (Glycolysis)	N/A
2– Deoxy-d-Glucose (2DG)	HK2 (Glycolysis)	NCT00096707 NCT00633087

Many metabolic inhibitors have been utilized in clinical trials, however few have led to FDA approval. This table provides a list of the inhibitors described in **Figure 3** and any associated clinical trials that have accepted lung cancer patients. N/A, not applicable.

Another molecule relied on by cancer is glutamine, which is transported into the cell through amino acid transporters including SLC1A5 (133). SLC1A5 is highly expressed in NSCLC cell lines and human tumor samples (133). Studies in NSCLC using the glutamine analogue L-γ-Glutamyl-p-nitroanilide (GPNA) revealed that glutamine is transported through SLC1A5 and that this transporter is required for proliferation in glutamine-dependent cell lines (A549, HCC15, and H520), which was confirmed using siRNA against SLC1A5 (**Table 2** and **Figure 3**) (133). Inhibition of SLC1A5 using GPNA also showed a marked increase in ROS generation due to a blockade of the glutamine to glutathione (ROS scavenger) conversion (133).

The last carbon source discussed is lactate, which is transported by MCT1/MCT4. Treatment with the MCT1 inhibitor SR13800 decreased lactate transport and enhanced OXPHOS in SCLC cell lines (**Table 2** and **Figure 3**) (134). Another small molecule inhibitor of MCT1, AZD3965, has been tested in SCLC cell lines and human-derived H526 xenograft models, which both exhibited a notable delay in cell proliferation and tumor growth, respectively (**Table 2** and **Figure 3**) (135, 136). Similarly, metabolites analyzed from *in vitro* experimentation with AZD3965 had alterations in amino acid and nucleotide biosynthesis and increased ROS generation (135), contributing to reduction in proliferation. Although a number of pathways are used by cancer cells, blocking the initial transport of carbon-source molecules have shown promise and deserve further attention of research in the treatment of lung cancer, where new treatment options are desperately needed to improve patient outcome and survival.

In addition to blockade of nutrient transport, inhibitors have been produced to target many enzymes in the glycolysis pathway, have high potential for efficacy, but have not been introduced into clinical practice as lung cancer therapies. When targeting the glycolysis pathway, the most well characterized inhibitor is 2DG (**Table 2** and **Figure 3**). In H23 human-derived NSCLC cells, treatment with 2DG inhibited cell growth and induced cell cycle arrest (57). Another study using human-derived H460 NSCLC cells found treatment with 2DG also activated PI3K/AKT signaling and phosphorylated Raf/MEK/ERK kinases, cell cycle and DNA damage molecules, and JAK/STAT proteins suggesting that the off target effects are far reaching and affect multiple pathways (137). Therefore, while promising, exploration into other enzymatic glycolysis inhibitors with fewer off targets would be more optimal. For that reason, inhibitors of PFKFB3 (rate-limiting enzyme of glycolysis) and LDHA (pyruvate to lactate converter) have been developed. Among the PFKFB3 inhibitors PFK-15 and the more potent PFK-158 have been the most encouraging and progressed into preclinical and clinical trials (**Table 2** and **Figure 3**) (38, 138). Unfortunately, these studies have not been conducted in NSCLC or SCLC models, however lung metastasis was reduced in head and neck squamous cell carcinoma (HNSCC) Cal27 xenografts treated with PFK-15 (139). Moreover, mesothelioma (a cancer that affects the pleural lining of the lungs and is linked to asbestos exposure) cells treated with PFK-158 exhibited reduced glycolysis and cell proliferation and this treatment alone was sufficient in reducing tumor growth without associated toxicities in xenograft mice (140). PFK-158 is currently undergoing clinical trial (NCT02044861) (141). Similarly to PFKFB3 inhibitors, several LDHA inhibitors have be produced, yet none of been extensively evaluated in preclinical or clinical trials. Although LDHA inhibition has not been previously examined, LDHA knockout NSCLC models have decreased tumor formation and even show regression of stablished tumors (142), providing evidence that LDHA may be a future viable target for lung cancer therapies.

Lastly, several reports on lung cancer metabolism suggest these tumors, particularly NSCLC, may be more oxidative, which provides an opportunity for metabolic intervention of mitochondrial respiration. Surprisingly, one of the most studied OXPHOS inhibitors in lung cancer is metformin, a common diabetes mellitus medication that blocks complex I of the ETC (**Table 2** and **Figure 3**) (42, 143). The anti-cancer activity of metformin has been documented in numerous cancer studies (143) and studies have found that diabetic patients with NSCLC on metformin even experience prolonged survival (144–146). While data investigating the therapeutic benefit of metformin in cancer may be encouraging, some evidence suggests that metformin use increases adaptive glycolysis activity (147), which would be counterproductive in metabolically-heterogeneous tumors and could increase therapy resistance. Additionally, it requires high dosing to achieve therapeutic advantage. Similar ETC complex I have been developed to overcome these drawbacks. phenformin, a structurally-similar anti-diabetic drug, was developed in an attempt to increase potency, however a therapeutic dose could not be achieved due to toxicity (**Table 2** and **Figure 3**) (42). Currently, a third ETC complex I inhibitor, IM156, with heightened potency and attainable therapeutic dosing is in Phase I clinical trial (**Table 2** and **Figure 3**) (148).

With the inherent metabolic nature of cancer, metabolism inhibitors are an underutilized category of therapy and should be considered as effective anti-cancer agents. Most metabolism-altering agents have displayed strong efficacy in cell lines and mouse models and those that have progressed into clinical trial, have been well tolerated. Further, metabolic inhibitors are actively taken in by the most metabolically active cells (i.e. the tumor) and therefore do not negatively affect cellular processes in non-malignant cells. With this knowledge future investigations of metabolic inhibition alone and in combination with the standard-of-care is essential for driving personalized lung cancer treatment options for all patients.

## Discussion

Cancer is an inherently metabolic disease, however cell origin, mutation status, oxygenation, and nutrient availability all contribute to the utilization of a particular metabolic program. To date, few metabolic inhibitors have progressed to clinical trial and those that have been clinically evaluated show moderate efficacy at best. Unfortunately, there has only been limited effort to metabolically characterize patient lung tumors or identify patients most likely to benefit. This is, in part, due to the difficulty of obtaining clinical samples since many lung cancers are not routinely surgically resected. Further difficulties may stem from the transient nature of metabolic pathway preference and differences between *in vitro* and *in vivo* cancer cells. These pitfalls highlight the urgency to identify viable biomarkers corresponding to the tumor metabolic profile.

**Figure 4:**
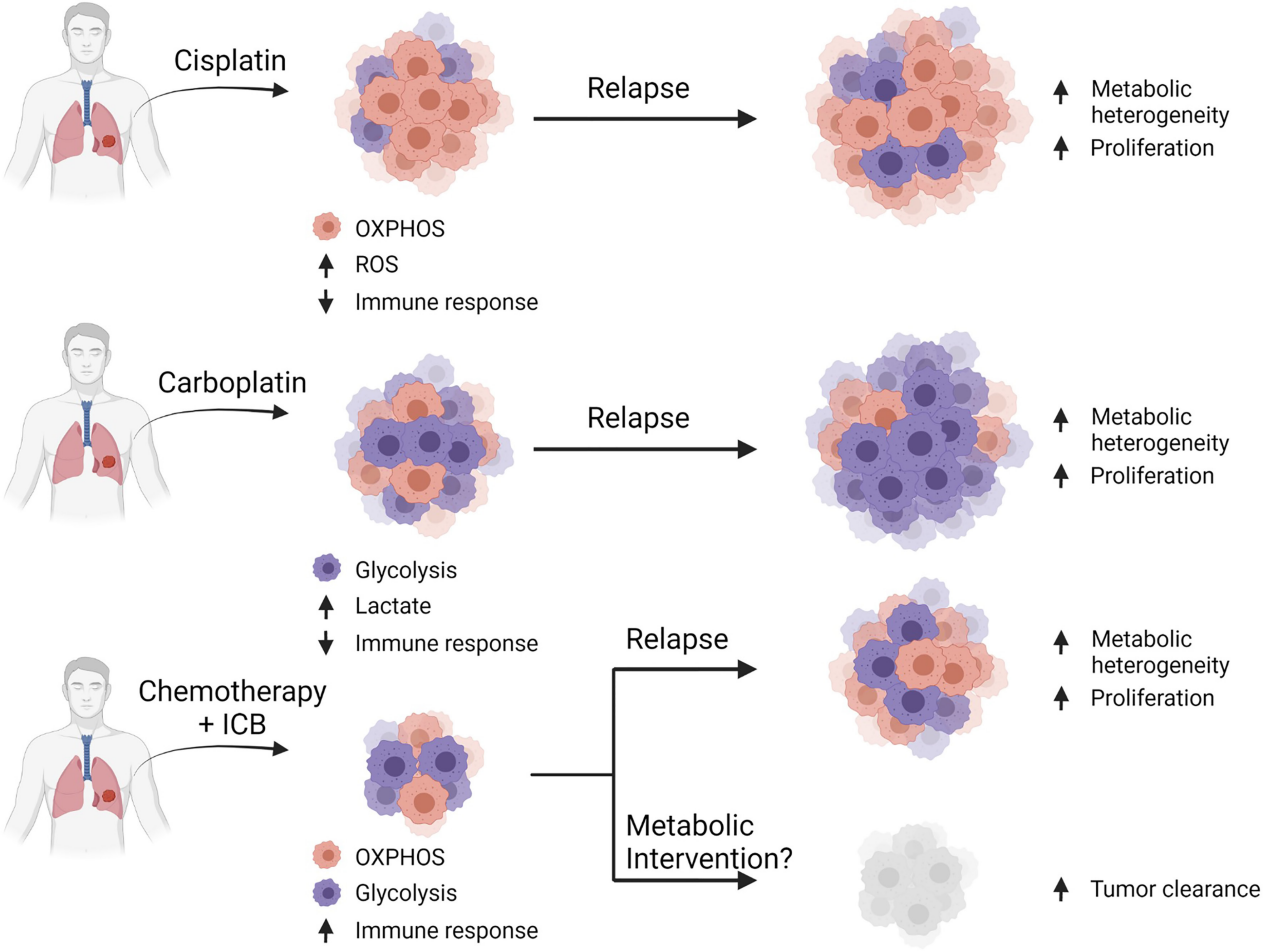
Combinatorial approaches for standard-of-care with metabolic inhibition. Hypothesis that common platinum-based chemotherapies select for cells dependent on their particular metabolic profiles. Cisplatin-based therapy selects for cells that are mostly oxidative, which leads to enhanced ROS production and reduced immune function leading to relapse. Carboplatin-based therapy selects for glycolytic cells leading to enhanced lactates production and reduced immune function resulting in relapse. A general chemotherapy plus immune checkpoint blockade provides some benefit in a few patients, but often ultimately leads to relapse, however metabolic intervention may provide additional efficacy by targeting pathways required for cellular proliferation.

We and others have previously shown that tumor heterogeneity exists in both NSCLC and SCLC and the administration of frontline treatment further exacerbates this phenotype (82, 149–151). It can be hypothesized that tumoral metabolism is also heterogenic, which would likewise enable clusters of glycolytic and oxidative cells that would become more profound after chemotherapy (**Figure 4**). For this reason, methods for patient metabolic phenotyping should be developed to assist with selecting the optimal combination of metabolic inhibitor in addition to frontline chemotherapy and ICB to delay tumor growth.

In conclusion, the effort to characterize lung cancer metabolism is at the forefront of investigation. There is ample evidence in support for targeting metabolic pathways to delay tumor growth as second-line single agents or in combination with frontline chemotherapy plus ICB. It is of utmost importance, however, to identify specific patient populations that would respond to such treatment efforts through biomarker analysis of cell surface or secreted molecules. Since cellular metabolism is a transient phenomenon, time course monitoring of identified biomarkers would be critical. If this can be achieved, the road will be paved for personalized therapies for targeted inhibition of metabolic pathways in lung cancer.

## Author Contributions

KC conceived and wrote the manuscript. WH wrote and edited the manuscript. KC and WH generated the figures and tables. LB and CG acquired funding and oversaw the writing and editing of the manuscript. All authors contributed to the article and approved the submitted version.

## Funding

The authors of this work are supported by the following funding agencies and fellowships: University of Texas SPORE in Lung Cancer P5-CA070907 (KC, CG, LB); NIH/NCI R01-CA207295 (LB); NIH/NCI U01-CA213273 (LB); NIH/NCI T32 CA009666 (CG); IALSC ILCF Fellowship (KC); ASCO Young Investigator Award (CG); The Khalifa Bin Zayed Al Nahyan Foundation (CG); through generous philanthropic contributions to The University of Texas MD Anderson Lung Cancer Moon Shot Program (LB)**;** The Andrew Sabin Family Fellowship (LB); CG and LB were supported by the Abell Hangar Foundation, the LUNGevity Foundation Career Development Award (CG), and the Rexanna Foundation for Fighting Lung Cancer (CG, LB).

## Conflict of Interest

CG serves in a consulting and advisory capacity for AstraZeneca, Kisoji Biotechnology, and Bristol Myers Squibb. CG is also a part of Speaker’s Bureau for AstraZeneca and Beigene. LB serves on advisory committees for AstraZeneca, AbbVie, GenMab, BergenBio, Pharma Mar SA, Sierra Oncology, Merck, Bristol Myers Squibb, Genentech, and Pfizer. LB also receives research support from AbbVie, AstraZeneca, GenMab, Sierra Oncology, Tolero Pharmaceuticals.

The remaining authors declare that the research was conducted in the absence of any commercial or financial relationships that could be construed as a potential conflict of interest.

## Publisher’s Note

All claims expressed in this article are solely those of the authors and do not necessarily represent those of their affiliated organizations, or those of the publisher, the editors and the reviewers. Any product that may be evaluated in this article, or claim that may be made by its manufacturer, is not guaranteed or endorsed by the publisher.
